# A novel costimulatory molecule gene-modified leukemia cell-derived exosome-targeted CD4^+^ T cell vaccine efficiently enhances anti-leukemia immunity

**DOI:** 10.3389/fimmu.2022.1043484

**Published:** 2022-11-16

**Authors:** Jiaqi Li, Fang Huang, Yan Jiang, Jie Zhao, Jiangbo Wan, Siguo Hao

**Affiliations:** Department of Hematology, Xinhua Hospital Affiliated to Shanghai Jiao Tong University School of Medicine, Shanghai, China

**Keywords:** CD4+ T cell, leukemia cells, costimulatory molecules (CD80 and CD86), gene modifiation, exosomes, tumor vaccine

## Abstract

Previous studies demonstrated that CD4^+^ T cells can uptake tumor antigen-pulsed dendritic cell-derived exosomes (DEXO), which harbor tumor antigen peptide/pMHC I complex and costimulatory molecules and show potent effects on inducing antitumor immunity. However, in preliminary study, CD4^+^ T cells targeted by leukemia cell-derived exosomes (LEXs) did not show the expected effects in inducing effective anti-leukemia immunity, indicating that LEX is poorly immunogenetic largely due to an inadequate costimulatory capacity. Therefore, LEX-based anti-leukemia vaccines need to be optimized. In this study, we constructed a novel LEX-based vaccine by combining CD4^+^ T cells with costimulatory molecules gene-modified LEXs, which harbor upregulated CD80 and CD86, and the anti-leukemia immunity of CD80 and CD86 gene-modified LEX-targeted CD4^+^ T cells was investigated. We used lentiviral vectors encoding CD80 and CD86 to successfully transduced the L1210 leukemia cells, and the expression of CD80 and CD86 was remarkably upregulated in leukemia cells. The LEXs highly expressing CD80 and CD86 were obtained from the supernatants of gene-transduced leukemia cells. Our data have shown that LEX-CD8086 could promote CD4^+^ T cell proliferation and Th1 cytokine secretion more efficiently than control LEXs. Moreover, CD4^+^ T_LEX-CD8086_ expressed the acquired exosomal costimulatory molecules. With acquired costimulatory molecules, CD4^+^ T_LEX-CD8086_ can act as APCs and are capable of directly stimulating the leukemia cell antigen-specific CD8^+^ CTL response. This response was higher in potency compared to that noted by the other formulations. Furthermore, the animal study revealed that the CD4^+^ T_LEX-CD8086_ significantly inhibited tumor growth and prolonged survival of tumor-bearing mice than other formulations did in both protective and therapeutic models. In conclusion, this study revealed that CD4^+^ T_LEX-CD8086_ could effectively induce more potential anti-leukemia immunity than LEX-CD8086 alone, suggesting that the utilization of a costimulatory molecule gene-modified leukemia cell-derived exosome-targeted CD4^+^ T cell vaccine may have promising potential for leukemia immunotherapy.

## Introduction

Although chemotherapy, targeted therapy, and hematopoietic stem cell transplantation (HSCT) have gone through substantial development during the last four decades, the prognosis of adult patients with relapsed/refractory leukemia remains poor; the 5-year overall survival (OS) rates for acute lymphoblastic leukemia (ALL) and acute myeloid leukemia (AML) are approximately 40% and 27%, respectively (1–4). Elderly patients with acute leukemia generally have worsened prognosis and show a 5-year OS rate of less than 20% (5). These findings emphasize the need for more effective treatment alternatives to improve the long-term outcome of leukemia, especially in elderly patients.

Immunotherapy has come to the fore in recent years (6, 7). It is known that tumor cells, including leukemia cells, can be recognized and destroyed by the immune system. New immunotherapies with chimeric antigen receptor (CAR) T cells, bi-specific T cell engagers (BiTEs), and immune checkpoint blockers (ICB) emerged as effective treatment options for chemo-resistant leukemia. However, many patients still show resistance to these immunotherapies due to leukemia intrinsic (e.g., target antigen loss, tumor heterogeneity) and extrinsic (e.g., immunosuppressive microenvironment) mechanisms driving treatment resistance (8–13). Although excellent results are seen with CAR-T cell therapy, this approach still shows a high disease recurrence largely due to T cell short time persistence and a lack of memory T cell formation (14, 15). Moreover, both CAR-T and BiTEs therapy face the challenge of the lack of a specific marker for AML blasts. Their current candidates showed widespread effects on non-AML healthy cells leading treatment-induced toxicity (4, 16). Furthermore, CAR-T therapy and BiTEs therapy have been associated with many toxicities (17). Adverse side-effects associated with CAR-T therapy and BiTEs therapy have been extensively reported with frequent events of cytokine release syndrome (CRS), driving a life-threatening multiple organ dysfunction syndromes, neurotoxicity, and the B cell aplasia (18). Therefore, CAR-T cells and BiTEs therapy for leukemia still fail to meet expectations. Interestingly, the direct and sustained activation of tumor-specific T cells *in vivo via* vaccination of anti-leukemia vaccines shows a superior therapeutic spectrum and safety profile. Anti-leukemia vaccines are being developed to disrupt tumor-associated tolerance, induce the expansion of tumor-specific effector cells, while maintaining immunomodulatory protection against autoimmunity. In addition, it may exert a synergistic antitumor effect with other immunotherapy such as CAR-T cells (19, 20).

Exosomes are small extracellular vesicles (40–200 nm) derived from late endosomes that mediate intercellular communication by shuttling lipids, proteins, and nucleic acids between cells. Their high bioavailability, biostability, biocompatibility, and cargo-loading suggests exosomes as a potential therapy for cancer patients (21–23). Many studies, including our previous efforts, proved the ability of tumor cell-derived exosomes (TEXs) and leukemia cell-derived exosomes (LEXs) to stimulate immune responses against cancer (24, 25). However, due to TEXs-induced immunosuppression and limited immunogenicity, the application of TEXs alone frequently results in an unsatisfying antitumor immunity *in vivo* (26).

Previous studies demonstrated that exosomal pMHC I and II complexes could be transferred to DCs, which activate T cells and lead to tumor eradication (27, 28). Therefore, the potential pathway of *in vivo* EXO-mediated antitumor immunity may be through up-take of EXO by host immature DC that, in turn, stimulate Ag-specific T lymphocytes *via* the pMHC complexes and costimulatory molecules on EXO-uptake DC. Our previous study demonstrated that mature DCs with the uptake of tumor antigen-pulsed DC-derived EXO express pMHC I and costimulatory CD40, CD54, and CD80 molecules and can strongly stimulate antigen-specific CD8^+^ CTL responses and antitumor immunity, and our later studies also demonstrated that mature DCs with the uptake of TEXs and LEXs could induce tumor or leukemia antigen-specific CD8^+^ CTL responses and anti-leukemia immunity (29–32). In addition, Kennedy et al. have demonstrated that CD4^+^ T cells can acquire antigen presenting cell (APC) membrane molecules *in vivo* and induce memory CTL responses (33). Our previous study also demonstrated that ovalbumin (OVA)-pulsed DC (DC_OVA_)-derived EXO (DEX_OVA_)-targeted active CD4^+^ T cells can stimulate central memory CD8^+^ CTL responses, induce more efficient antitumor immunity and T cell memory than DC_OVA_, and counteract regulatory T cell-mediated immune suppression (34). Therefore, these results indicate that DC-derived EXO (DEX) can transfer DC’s Ag, presenting the activity to either DC or CD4^+^ T cells through EXO uptake.

Then, whether LEXs enriched of leukemia cell antigens could target CD4^+^ T cells and LEXs-targeted CD4^+^ T cells could induce strong anti-leukemia immunity or not was still unclear? In a preliminary study, our data showed that, unlike DEX, LEXs-targeted CD4^+^ T cells could not induce an efficiently anti-leukemia immune response. Therefore, we conjecture that lower level expression of costimulatory molecules on LEXs than that on DEX may be the main defect for LEXs-targeted CD4^+^ T cells to induce high-level anti-leukemia immunity, since costimulatory molecule gene-modified LEXs could significantly enhance their anti-leukemia immunity in our recent study (35). Other studies also demonstrated that uptake of TEXs by DCs might enhance the expression levels of costimulatory molecules (CD80 and CD86) and prime immune responses (36–38). In this study, we collected LEXs derived from mouse leukemia L1210 cells transduced with a lentiviral vector encoding CD80 and CD86 and investigated the anti-leukemia immunity of LEX-CD8086-targeted CD4^+^ T cells *in vitro* and *in vivo*.

## Methods

### Materials and regents

RPMI 1640 medium, Dulbecco’s Modified Eagle’s Medium (DMEM), Penicillin and Streptomycin, fetal bovine serum (FBS), and Dynabeads Mouse T-Activator CD3 and CD28 beads were purchased from Gibco BRL/Life Technologies (Grand Island, NY, USA). IL-2 (212–12–5) was provided by PeproTech (Rocky Hill, NJ, USA). Exosome-depleted FBS was obtained from System Biosciences (Mountain View, CA, USA). CFSE and LDH cytotoxicity assay kit purchased from Invitrogen (Shanghai, China). Rabbit anti-mouse TSG101 (ab125011), CD63 (ab217345), CD9 (ab92726) and CD81 (ab109201) were purchased from Abcam (Shanghai, China). Rabbit anti-mouse HSP70 (4872) and Ki-67 (12202) were purchased from Cell Signaling Technology (Shanghai, China). Anti-GAPDH (GB11002) was provided by Servicebio (Wuhan, Hubei, China). Anti-mouse CD4 PE (12-0041-81), anti-mouse CD8a APC (17-0081-81), Anti-Mouse CD69 FITC (11-0691-82), anti-mouse/Rat Ki67 PE (12-5698-80), and Fixable Viability Dye eFluor 780 (65-0865-14) used for flow cytometry analysis were purchased from eBioscience (San Diego, CA, USA). Anti-mouse CD4-APC (100515), anti-mouse CD86-APC (105011), anti-human/mouse Granzyme B-PE (372208), anti-mouse FITC-CD8(100706) and anti-mouse Perforin-APC (154404) were purchased from Biolegend (San Diego, CA, USA). Anti-mouse CD80-PE (561955), 7-AAD staining solution (559925), and the Cytometric Bead Array (CBA) Mouse Th1/Th2 Cytokine Kit were obtained from BD Biosciences (San Diego, CA, USA).

### Cell line and animals

The murine lymphocytic leukemia cell lines L1210-null and L1210-CD8086 were cultured in DMEM medium supplemented with 10% (v/v) FBS, penicillin, and streptomycin (1%). In addition, p388 cells were cultured in RPMI 1640 supplemented with 10% (v/v) FBS, penicillin, and streptomycin (1%). Cells were cultured at 37°C in an incubator containing 5% CO_2_. L1210 and p388 cells were purchased from the Shanghai Institute for Biological Science (Shanghai, China).

Six to eight-week-old DBA/2 female mice were purchased from the Shanghai SLAC Laboratory Animal Center (Shanghai, China) and kept under specific, pathogen-free (SPF) conditions. According to the Ethics Committee of the Xinhua Hospital guidelines, all animal experiments were conducted at the animal center in Xinhua Hospital Affiliated to Shanghai Jiao Tong University School of Medicine, Shanghai, China.

### Construction of a L1210 cell line stably overexpressing CD80 and CD86

To produce the leukemia cells that highly express CD80 and CD86, L1210 cells were transduced with recombinant lentiviral vectors encoding CD80 and CD86 genes and selected by 2 μg/ml puromycin for 72 h as previously mentioned (35). Briefly, the lentiviral vectors expressing the green fluorescence and anti-puromycin proteins were established in our laboratory and stored at −80°C. Fluorescence intensity and anti-puromycin screening tests were used to ensure virus titration and lentiviral infection. When the inverted fluorescence microscope showed that the cells were all stained with green fluorescence, we concluded that stable L1210 cells over-expressing CD80 and CD86 were constructed successfully. These constructed L1210 leukemia cells were termed L1210-CD8086. The short hair (sh) RNA sequence for gene over-expression was used as follows: Mouse EF1-CD80 Forward: AGCTGTGACCGGCGCCTACATGGCTTGCAATTGTCAGTTG; Mouse EF1-CD80 Reverse: AAGGAAGACGGTCTGTTCAGC; Mouse CD86 Forward: ATGGACCCCA GATGCACCAT; Mouse CD86 Reverse: TCACTCTGCATTTGGTTTTG.

### Real-time PCR

Total RNA was extracted using an RNA purification kit (EZBioscience, Roseville, MN, USA) and was reverse-transcribed into cDNA using the Primescript RT Master Mix (Takara, Beijing, China) following the manufacturers’ instructions. Real-time PCR was performed using the SYBR Premix Ex Taq (Yeasen, Shanghai, China) and specific primers in the Applied Biosystems™ QuantStudio™ 3 Real-Time PCR system. The PCR conditions were one cycle at 95°C for 5 min and 40 cycles of 10 s at 95°C and 30 s at 60°C. *GAPDH* was used as an internal control. The data were analyzed by 2-DDCt. Primers for real-time PCR were used as follows: Mouse CD80 Forward: ACCCCCAACAT AACTGAGTCT; Mouse CD80 Reverse: TTCCAACCAAGAGAAGCGAGG; Mouse CD86 Forward: ATGGACCCCAGATGCACCAT; Mouse CD86 Reverse: TCACTCTGC ATTTGGTTTTG; Mouse GAPDH Forward: GGTTGTCTCCTGCGACTTCA; Mouse GAPDH Reverse: TGGTCCAGGGTT TCTTACTCC.

### Exosome preparation

To obtain the exosomes derived from L1210-CD8086 cells, L1210-CD8086 cells were pre-cultured in a complete medium containing 10% exosome-free FBS for 24 h to avoid contamination from the serum, after which the culture supernatants were collected for the isolation of exosomes as reported previously (31). Briefly, culture supernatants were centrifuged at 300 ×g for 10 min, then at 2,000 ×g for 20 min, and 10,000 ×g for 30 min to eliminate cells and debris. Next, the supernatants were ultracentrifuged at 100,000 ×g for 70 min at 4°C to pellet the exosomes. Finally, the exosome pellets were washed in a large volume of PBS and were recovered by centrifugation at 100,000 ×g for 70 min. The BCA assay measured the exosomal proteins (Beyotime Biotech, Shanghai, China) were stored at −80°C. The exosomes purified from the supernatants of L1210-CD8086 were named LEX-CD8086, and LEXs purified from the supernatant of L1210 cells transduced with a null vector were named LEX-null.

### Western blot

A total of 20 μg exosomal proteins were separated using 12% SDS-polyacrylamide gel electrophoresis and transferred onto PVDF membranes. The blots were blocked with 5% non-fat dry milk at room temperature for 1 h and incubated overnight at 4°C with the corresponding primary antibodies at dilutions recommended by the suppliers, followed by incubation with HRP-conjugated secondary antibodies (Beyotime Biotech, Shanghai, China) at room temperature for 1 h. The blots on the membranes were visualized using enhanced chemiluminescent reagents (Thermo Fisher Scientific, Shanghai, China). CD63, CD9, HSP70, CD81 and TSG101 were used as exosome markers. GAPDH was used as a loading control.

### Electron microscopy and nanoparticle analysis

Exosomes were placed on 200-mesh carbon-coated copper grids at RT for 2 min. The excess suspension was removed using filter paper. The exosomes were negatively stained with uranyl acetate at RT for 5 min, washed twice with PBS, and dried. The exosomes were examined using a Philips CM12 transmission electron microscope operating at 80 kV, and their images were captured. The size distribution of exosomes was analyzed using the qNano Gold Particle Sizing Instrument (Izon Science, OX, UK).

### Flow cytometry

For exosome flow cytometry analysis, 30 μg of exosomes were coated onto 4 μm Aldehyde/sulfate latex microbeads (Invitrogen, Eugene, OR, USA), as described previously (39). The exosome-coated beads were stained with the corresponding fluorescence-labeled Abs for 30 min on ice. Next, cells were washed with PBS twice and incubated with the corresponding fluorescence Abs for 30 min on ice for cell surface staining. For intracellular staining, cellular staining was performed for 60 min on ice after using a fixation/permeabilization kit (eBioscience, San Diego, CA, USA). Finally, after being washed with PBS twice, all the events were harvested and analyzed by flow cytometry. Data were analyzed using FlowJo software (Tree Star, Ashland, OR, USA).

### CD4^+^ T cell preparation

Splenic CD4^+^ T cells were isolated from DBA/2 mice spleens, enriched by passage through nylon wool columns, and then purified using EasySep™ mouse CD4^+^ T-cell isolation kit according to the manufacturer’s instructions (Stem cell Technologies, Vancouver, Canada) to yield populations that were >95% CD4^+^ T cells. To generate activated CD4^+^ T cells, the splenic CD4^+^ T cells were cultured in a 10% FBS RPMI 1640 medium containing IL-2 (100 IU/ml) and activated by mouse T-activator CD3 and CD28 beads (25 μL/ml) for 24 h.

### Exosomes were taken up by CD4^+^ T cells

To verify the physical interactions between LEX-CD8086 and CD4^+^ T cells, the LEX-CD8086 were stained with 5 μM CFSE in 100 µl PBS for 20 min, then washed with 10 ml PBS and pelleted by ultracentrifugation. First, the CD4^+^ T cells were incubated with CFSE-labeled LEX [25 μg/1×10^6^ T cells in 200 μl of medium containing IL-2 (100 IU/ml)] at 37°C for 4 to 24 h and analyzed by immunofluorescence microscopy every 2 to 4 h. When the green fluorescence of the CD4^+^ T cells reached a maximal level (85% CFSE-positive cells), the CD4^+^ T cells were harvested and the Nuclei of CD4^+^ T cells were stained with DAPI. Samples were observed using a Nikon confocal microscope. In another set of experiments, the CD4^+^ T cells were incubated with LEX-CD8086 under the above conditions for 6 h, and then the expression of CFSE, CD69, CD80 and CD86 on CD4^+^ T cells was assessed by flow cytometry. The CD4^+^ T cells incubated with LEX-CD8086 were named CD4^+^ T_LEX-CD8086_ cells, and the CD4^+^ T cells incubated with LEX-null were named CD4^+^ T_LEX-null_ cells.

### Cytotoxicity assay

To assess the functional effect of CD4^+^ T_LEX-CD8086_ cells, we performed a cytotoxicity assay to examine whether CD4^+^ T_LEX-CD8086_ cell vaccination could induce an efficient anti-leukemia cytotoxic T lymphocyte response. Briefly, splenic CD8^+^ T cells were isolated from mice immunized with PBS, LEX-null (30 μg/mouse), LEX-CD8086 (30 μg/mouse), CD4^+^ T cells (1×10^6^/mouse), CD4^+^ T_LEX-CD8086_ cells (1×10^6^/mouse), or CD4^+^ T_LEX-null_ cells (1×10^6^/mouse). Seven days after the last stimulation, splenic CD8^+^ T cells were isolated from immunized mice using an EasySep™ mouse CD8^+^ T cell Isolation Kit (Stem-cell Technologies, Vancouver, Canada). Cells were re-stimulated with irradiated L1210 cells for seven days and then harvested as effector cells. L1210 cells were used as specific target cells, and p388 cells were used as controls. The corresponding target cells (1 × 10^4^/mL) were mixed at different ratios with effector cells overnight at 37°C and detected by an LDH Cytotoxicity Assay kit (Invitrogen, Eugene, OR, USA) according to the manufacturer’s instructions. The spontaneous/maximal release ratio in all experiments was lower than 20% (<20%). Therefore, the specific lysis was calculated as follows: (experimental LDH release − effector cells − target spontaneous LDH release)/(target maximum LDH release) × 100 [48].

### Animal studies

To evaluate the protective immunity of CD4^+^ T_LEX-CD8086_ cells against leukemia cells, DBA/2 mice were immunized by subcutaneous (s.c.) injection at the inner side of the right hind thighs with PBS, LEX-null (30 μg/mouse), LEX-CD8086 (30 μg/mouse), CD4^+^ T cells (1×10^6^/mouse), CD4^+^ T_LEX-CD8086_ cells (1×10^6^/mouse), or CD4^+^ T_LEX-null_ cells (1×10^6^/mouse). The injected mice were boosted twice with the above vaccine formulations for a 7-day interval. Following seven days of the last immunization, the mice were s.c. challenged with L1210 cells (2× 10^5^ cells/mouse) to the outer side of the same thighs, and the tumor growth was monitored daily. Tumors were measured using a digital caliper; tumor volume was calculated as length × [width] ^2^ × 0.50. Mice bearing subcutaneous tumors with diameters reaching 20 mm were euthanized according to the regulations of the Shanghai Jiao Tong University Laboratory Animal Unit and counted as dead. Immediately following euthanasia, blood samples were harvested from the eyeball and detected by the mouse Th1/Th2 Cytometric Bead Array (CBA) kit following the manufacturer’s instructions. Next, spleen and tumor samples were harvested for flow cytometric analysis. Next, single-cell suspensions were prepared, and red blood cells were lysed using ACK Lysis Buffer. Finally, the tumor was dissected for immunohistochemistry (IHC) and flow cytometric analysis.

To examine the therapeutic effects on the established tumors, L1210 cells (2 × 10^5^ cells/mouse) were injected into the outer side of the right thighs. When the tumors became palpable (generally on the 7th day, with approximately 3.5 mm in diameter), an injection of the vaccines mentioned above was administered to the inner side of the thighs of the inoculated mice. The therapy was performed thrice at a 3-day interval each time, and the tumor growth was monitored every two days. Mice bearing subcutaneous tumors with diameters reaching 20 mm were euthanized according to the regulations of the Shanghai Jiao Tong University Laboratory Animal Unit and counted as dead. Tumor volume was calculated as length × [width]^2^ × 0.50. The spleen, lymph nodes, and tumors were harvested for flow cytometry. A single-cell suspension was prepared, and red blood cells were lysed using ACK Lysis Buffer.

### Immunohistochemistry analysis

Tumor were dissected out and fixed with 10% paraformaldehyde and embedded in paraffin. Sections were deparaffinized and incubated with antibodies against Ki-67 (Cell Signaling Technology) followed by visualization with the one‐step polymer detection system (ZSGB‐bio company, Beijing, China). To visualize the expression of Ki-67, images of tumor tissue were captured using a microscope with CCD.

### Statistical analysis

Experiments were performed at least three times. Statistical analyses were performed using GraphPad Prism software. Data are presented as the mean ± SD. Differences between two groups were evaluated by Student’s t test, and differences among three or more groups were analyzed with ANOVA. Survival data were analyzed by the long-rank test. Differences between samples were considered statistically significant when P < 0.05.

## Results

### LEXs harboring high levels of CD80 and CD86 were obtained from the costimulatory molecule gene-modified L1210 cells

**Figure 1:**
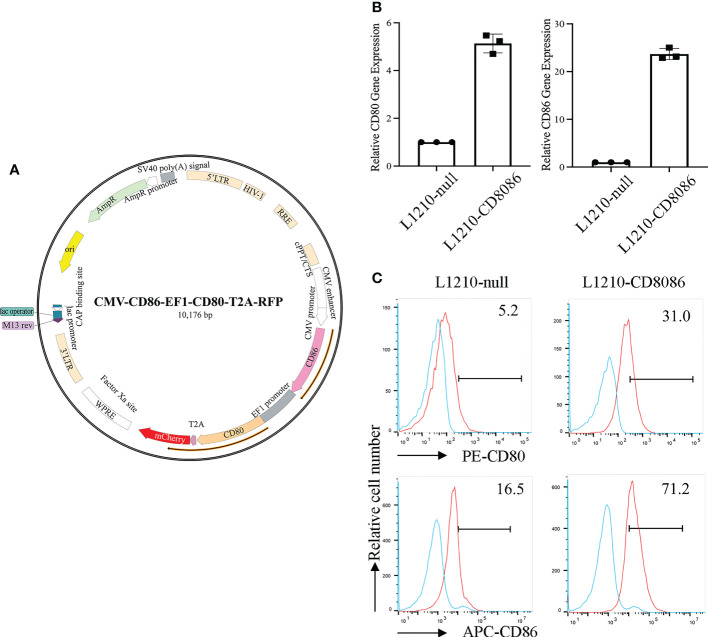
Construction of a lentiviral vector encoding costimulatory molecules and transduction of the L1210 cells. **(A)** The map of a lentiviral vector encoding CD80 and CD86. **(B)** The expression of CD80 and CD86 in L1210-null and L1210-CD8086 cells was quantified by real-time PCR and flow cytometry **(C)**. Data in **(B)** are presented as mean ± SD. P values are evaluated by Student’s t test.

**Figure 2:**
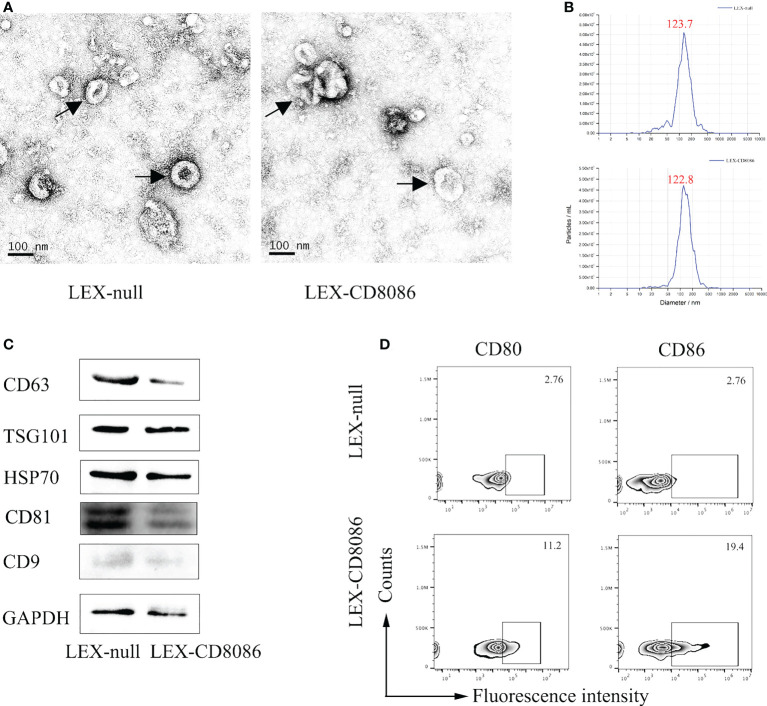
Characterization of exosomes derived from LEX-CD8086 cells. **(A)** The morphology of the LEX derived from of L1210-null cell (LEX-null, Left) or L1210-CD8086 cell (LEX-CD8086, Right) determined by transmission electron microscopy. Black arrow indicates exosome. **(B)** Size distribution of LEX. The size distribution of exosomes determined by nanoparticle tracking. Peaks at 123.7 nm (LEX-null, above) and 122.8 nm (LEX-CD8086, below). **(C)** The exosomal protein markers of LEX-CD8086 and LEX-null were detected by western blotting. **(D)** The CD80 and CD86 expressions of LEX-CD8086 were determined by flow cytometry, LEX-null was used as the control.

To upregulate the expression of costimulatory molecules on leukemia cells, we constructed a lentiviral vector encoding the CD80 and CD86 genes (35). The L1210 cells were transduced with this lentiviral vector (**Figure 1A**). After transduction, CD80 and CD86 expression in L1210 cells was markedly increased (**Figures 1B, C**). To produce the LEXs derived from CD80 and CD86 gene-modified L1210 cells (L1210-CD8086), L1210-CD8086 cells were cultured in an exosome-free medium for 24 hours. The conditioned medium was then collected and subjected to differential ultracentrifugation. To examine whether gene transduction would affect the biology of LEXs, we examined and characterized the LEXs derived from L1210-CD8086 cells (LEX-CD8086) using electron microscopy and nanoparticle tracking analysis (NTA). Our data showed that the LEX-CD8086 were physically homogeneous, exhibited the dimpled, cup-shaped characteristic morphology, and were in a size range of ~40 to 160 nm in diameter (**Figures 2A, B**). Furthermore, western blot analysis indicated that the LEX-CD8086 fully expressed HSP70, CD9, CD63, CD81 and TSG101, which are considered typical exosomal proteins (**Figure 2C**) and were negative for the endoplasmic reticulum protein GRP94 (data not showed). Meanwhile, the expression of CD80 and CD86 in LEX-CD8086 was significantly upregulated compared to that in LEX-null (**Figure 2D**). Altogether, these data suggest that obtaining LEX highly expressing CD80 and CD86 through the costimulatory molecule gene-modified leukemia cells is feasible.

### LEX-CD8086 can be taken up by CD4^+^ T cells and transfer exosomal CD80 and CD86 to CD4^+^ T cells

**Figure 3:**
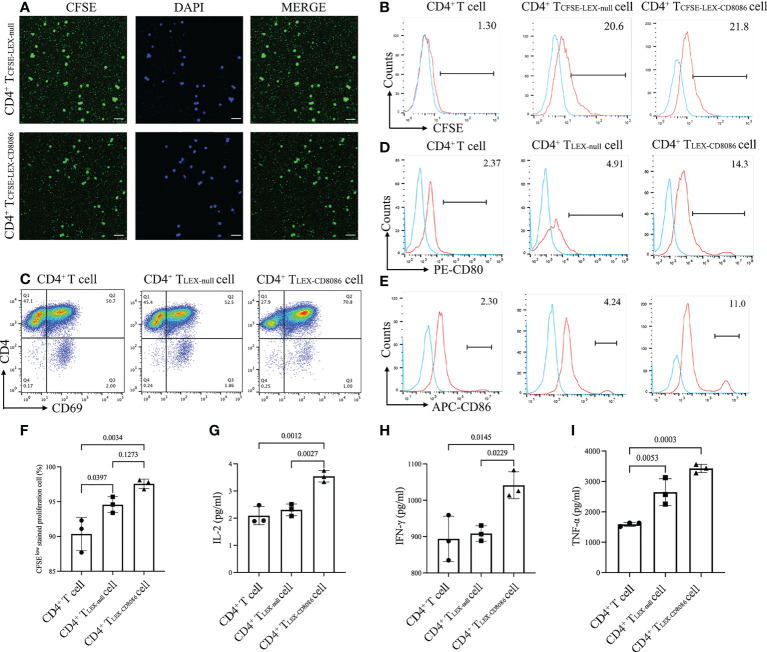
LEX-CD8086 uptake by CD4^+^ T cells and the effects of LEX-CD8086 on CD4^+^ T cell proliferation and cytokine assay. **(A)** Fluorescence microscope analysis of mouse splenic CD4^+^ T cells (stimulated with anti-CD3/CD28 antibodies) after incubation with CFSE-labeled L1210-CD8086 or L1210-null cell-derived exosomes for 6 h. bar scale = 20 μm. **(B)** Flow cytometric analysis of the cellular uptake of mouse splenic CD4^+^ T cells after incubation with CFSE-labeled L1210-CD8086 or L1210-null cell-derived exosomes for 6 h. **(C–E)** Analysis of CD69, CD80 and CD86 expression on CD4^+^ T cells after co-cultured with LEX-CD8086. **(F)** The proportion of CFSE^Low stained^ CD4^+^ T cells after incubation with LEX-CD8086 by flow cytometry and quantitative analysis of the results of CFSE^Low stained^ CD4^+^ T cells. Secretion of IL-2 **(G)**, IFN-γ **(H)**, and TNF-α **(I)** in the supernatants of CD4^+^ T cells cocultured with LEX-CD8086. All the results shown above represent at least three independent experiments. Data are mean ± SD of three independent biological replicates. P values are from ANOVA.

Our previous studies demonstrated that CD4^+^ T cells can uptake tumor antigen and antigen-presenting molecules from dendritic cells-derived exosomes and inducing antitumor immunity. To assess whether LEX could be uptake by CD4^+^ T cells, CD4^+^ T cells were first incubated with CFSE-labeled LEX (LEX_CFSE_) and then analyzed by flow cytometry and confocal fluorescence microscopy. Our data showed that the uptake of LEX_CFSE_ by CD4^+^ T cells increased with incubation time and reached a maximal level (85% CFSE-positive cells) after 6 h incubation, which was also confirmed by confocal fluorescence microscopic analysis and flow cytometry (**Figures 3A, B**). To investigate CD4^+^ T cell uptake of exosomal costimulatory molecules and activated by LEX-CD8086, CD4^+^ T cells were incubated with LEX-CD8086 for 6 hours, and the expression of CD69, CD80 and CD86 on CD4^+^ T cells was analyzed by flow cytometry. As shown in **Figures 3C–E**, the expression of CD69, CD80 and CD86 in LEX-CD8086-targeted CD4^+^ T cell (CD4^+^ T_LEX-CD8086_ cell) was significantly higher than in LEX-null-targeted CD4^+^ T cell (CD4^+^ T_LEX-null_ cells; p < 0.05), indicating that exosomal costimulatory molecules can be transferred from LEX-CD8086 to CD4^+^ T cells, which could make CD4^+^ T cells act as APCs. Meanwhile, our data also showed that LEX-CD8086 can promote CD4^+^ T cell proliferation (**Figure 3F**) and secretion of IFN-γ, TNF-α, and IL-2 during co-cultured with LEX-CD8086 (**Figures 3G–I**), which are recognized as indicators of a Th1 response. Taken together, our data suggest that LEX-CD8086 can be taken up by CD4^+^ T cells and transfer their exosomal costimulatory molecules to CD4^+^ T cells, and LEX-CD8086 can promote CD4^+^ T cells differentiation into Th1 cells.

### CD4^+^ T_LEX-CD8086_ cells can induce efficient antigen-specific anti-leukemic CTL response

**Figure 4:**
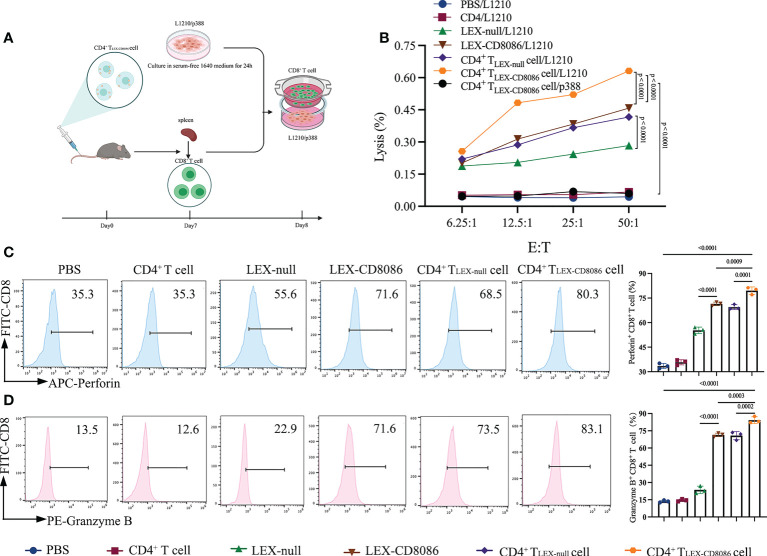
Cytotoxicity assay. **(A)** Schematic protocol of CD8^+^ T cells isolation from mice immunized with CD4^+^ T_LEX-CD8086_ cells. **(B)** Cytotoxicity assay. The cytotoxic activity of L1210 cells of CD8^+^ T cells from mice immunized with PBS, CD4^+^ T cells, LEX-null, LEX-CD8086, CD4^+^ T_LEX-null_ cells, and CD4^+^ T_LEX-CD8086_ cells as assessed by LDH cytotoxicity assay at the indicated effector-to-target (E: T) ratios. The separated viable CD8^+^ T cells served as effector cells. L1210 or p388 cells served as target cells. **(C)** Analysis of Perforin expression of CD8^+^ T cells from mice immunized with the vaccinations as mentioned above (Left) and quantitative analysis of the results of expression of Perforin (Right). **(D)** Analysis of Granzyme B expression of CD8^+^ T from mice immunized with the vaccinations as mentioned above formulations **(**Left**)** and quantitative analysis of the results of expression of Granzyme B **(**Right**)**. All the results shown above represent at least three independent experiments. Data are mean ± SD of three independent biological replicates. P values are from ANOVA.

To determine the functional effect of CD4^+^ T _LEX-CD8086_ cells on the ability to induce CD8^+^ T cells to anti-leukemia, DBA/2 mice were immunized with LEX-null, LEX-CD8086, CD4^+^ T_LEX-null_ cells and CD4^+^ T_LEX-CD8086_ cells and with PBS, CD4^+^ T cells as control, and then harvested the splenic CD8^+^ T cells (**Figure 4A**). First, we performed the cytotoxicity assay. As shown in **Figure 4B**, the splenic CD8^+^ T cells derived from the CD4^+^ T_LEX-CD8086_ cells immunized mice displayed the most strong cytotoxic activity versus L1210 cells (63.1% killing; E/T ratio, 50:1) compared with that from CD4^+^ T_LEX-null_ cells (51.5%) and LEX-CD8086 (48.5%) immunized mice (p < 0.0001), the splenic CD8^+^ T cells derived from the control mice did not have any killing activity against L1210 cells; Besides, the splenic CD8^+^ T cells stimulated with CD4^+^ T_LEX-CD8086_ cells did not show any killing activity against p388 cells, indicating that the anti-leukemia CTL response induced by CD4^+^ T_LEX-CD8086_ cells is leukemia cell antigen-specific. Moreover, we also examined Perforin and Granzyme expression of splenic CD8^+^ T cells derived from above mentioned immunized mice. As shown in **Figures 4C, D**, the percentage of splenic CD8^+^ T cells expressing Perforin and Granzyme in CD4^+^ T _LEX-CD8086_ cells immunized mice was 79.5% and 84.3%, respectively, which were significantly higher than that in mice immunized with CD4^+^ T_LEX-null_ cells (69.5% and 70.8%), LEX-CD8086 (71.40% and 71.43%) and LEX-null (55.30% and 23.60%). Altogether, our data suggest that CD4^+^ T_LEX-CD8086_ cells can potentially induce a stronger leukemia antigen-specific anti-leukemia CTL immune response than CD4^+^ T_LEX-null_ cells and LEX-CD8086 alone.

### CD4^+^ T_LEX-CD8086_ cells can induce protective immunity against leukemia cells

**Figure 5:**
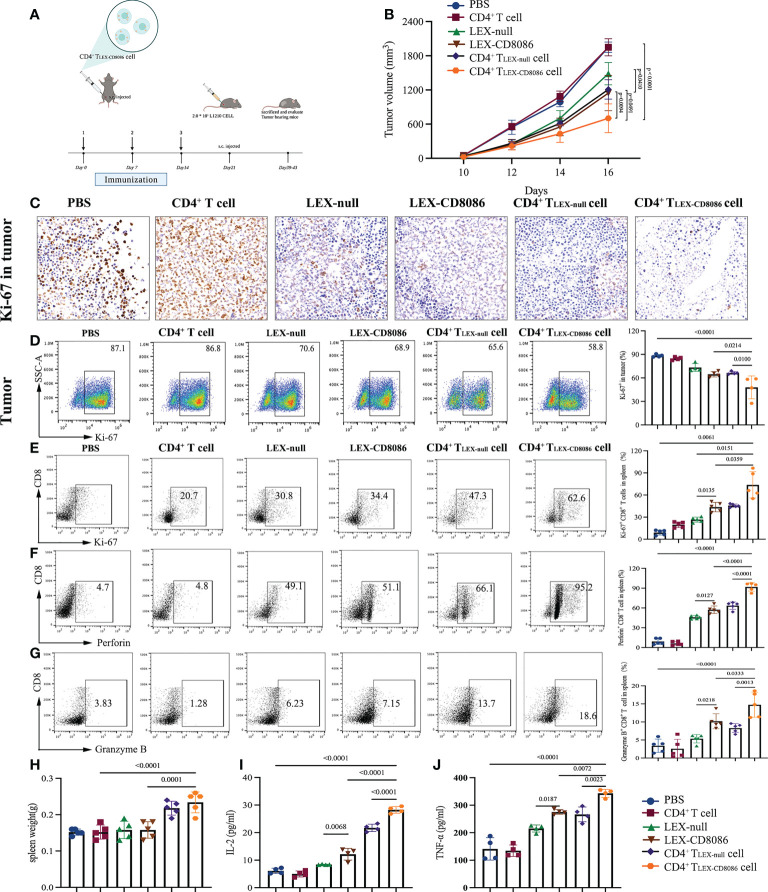
CD4^+^ T_LEX-CD8086_ cell immunization induces potent anti-leukemia preventive immunity. **(A)** The schematic protocols of the experimental design in an animal study. The DBA/2 mice were pre-injected with PBS, CD4^+^ T cells, LEX-null, LEX-CD8086, CD4^+^ T_LEX-null_ cells, and CD4^+^ T_LEX-CD8086_ cells on Days 0, 7, and 14 and then injected with L1210 cells on Day 21 and sacrificed when tumor diameter reached 20 mm. **(B)** Tumor volume was measured after tumor challenge with calipers. **(C)** After the indicated vaccinations, representative immunohistochemistry images of Ki-67 expression in L1210 tumor tissues. **(D)** Analysis of Ki-67 expression in tumor tissues after the indicated vaccinations (Left). Quantitative analysis of the results of Ki-67 expression in tumor tissues (Right). **(E–H)** Flow cytometry analysis of expression of Ki-67, Perforin,and Granzyme B in CD8^+^ T cells in the spleens from mice immunized with the vaccine as mentioned above (Left) and quantitative analysis of the results of expression of Ki-67, Perforin,and Granzyme B in CD8^+^ T cells in the spleens of the mice (Right). **(G)** The spleen weight of mice at the end of vaccinations. **(I, J)** Detection of IL-2 and TNF-α in the serum of mice at the end of vaccinations. All data in this figure are presented as means ± SD. P values are from ANOVA.

To examine whether CD4^+^ T_LEX-CD8086_ cells can induce the protective immune against leukemia cells *in vivo*, DBA/2 mice were immunized with LEX-null, LEX-CD8086, CD4^+^ T_LEX-null_ cells, CD4^+^ T_LEX-CD8086_ cells, and PBS, as a control, for three times, and the mice were then subcutaneously (s.c.) challenged with L1210 cells seven days after the last vaccination. Tumor growth and survival were monitored and recorded (**Figure 5A**). Immunization with CD4^+^ T_LEX-CD8086_ cells significantly inhibited tumor growth compared with that in mice immunized CD4^+^ T_LEX-null_ cells and LEX-CD8086. As shown in **Figure 5B**, at Day 16, the mean tumor volume in CD4^+^ T_LEX-CD8086_ cell immunized mice (703 mm^3^) was significantly smaller than that in CD4^+^ T_LEX-null_ cell (1209 mm^3^), LEX-CD8086 (1146 mm^3^), LEX-null (1552 mm^3^) immunized mice, all the mice in the PBS and CD4^+^ T cells control group died 20 days after the tumor cell challenge. In addition, immune-histochemistry (**Figure 5C**) and flow cytometry (**Figure 5D**) analysis also showed that the Ki-67 expression of the tumor tissues in CD4^+^ T_LEX-CD8086_ cells immunized mice was 58.8%, which was significantly lower than that in mice immunized with CD4^+^ T_LEX-null_ cells (65.6%), LEX-CD8086 (68.9%) and LEX-null (70.6%). Moreover, we also assessed the expression of Ki-67, Perforin, and Granzyme of CD8^+^ T cells in the spleen of the above-immunized mice. As shown in **Figures 5E–G**, the proportion of splenic CD8^+^ T cells expressing Ki-67, Perforin, and Granzyme B in CD4^+^ T_LEX-CD8086_ cell immunized mice were 62.6%, 95.2%, and 18.6%, respectively, which were significantly higher than those in mice immunized with CD4^+^ T_LEX-null_ cell (47.3%, 66.1%, and 13.7%), LEX-CD8086 (34.4%, 51.1%, and 7.15%), LEX-null (30.8%, 49.1% and 6.23%) and controls. Additionally, the weight of the spleen of the mice immunized with CD4^+^ T_LEX-CD8086_ cells was the heaviest among the six groups (**Figure 5H**), indicating that CD4^+^ T_LEX-CD8086_ cells may efficiently activate antitumor immunity systemically. Meanwhile, we also examined the circulating Th1 cytokines in the peripheral blood of all immunized mice. As shown in **Figures 5I, J**, the levels of IL-2 (28.3 pg/ml) and TNF-α (343.2 pg/ml) in the serum of mice immunized with CD4^+^T_LEX-CD8086_ cells were notably higher than those in mice immunized with CD4^+^ T_LEX-null_ cells (21.7 pg/ml, 266.5 pg/ml), LEX-CD8086 (12.2 pg/ml, 275.5 pg/ml), LEX-null (8.4 pg/ml, 215.2 pg/ml), CD4^+^ T cell (5.0 pg/ml, 134.6 pg/ml), and PBS (6.5 pg/ml, 141.2 pg/ml). Altogether, our data suggest that CD4^+^ T_LEX-CD8086_ cells can induce the most potential antitumor protective immunity.

### CD4^+^ T_LEX-CD8086_ cells induced potent therapeutic efficacy

**Figure 6:**
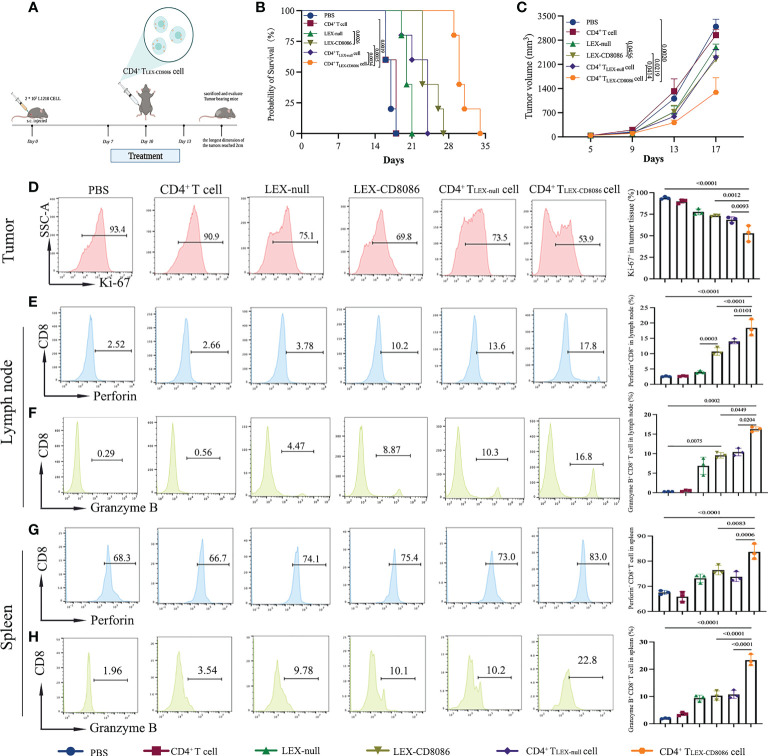
CD4^+^ T_LEX-CD8086_ cell immunization induces potent anti-leukemia therapeutic immunity. **(A)** Schematic protocols of the experimental design in the animal study. DBA/2 mice were injected with L1210 cells. When the tumors became palpable (generally, on the 7th day), the mice were s.c. injected with different vaccine formulations, including CD4^+^ T cells, LEX-nulls, LEX-CD8086, CD4^+^ T_LEX-null_ cells, and CD4^+^ T _LEX-CD8086_ cells on Day7, 10, and 13. **(B)** Mice survival. **(C)** Tumor growth. **(D)** Analysis of Ki-67 expression in tumor tissues after the indicated treatments (Left). Quantitative analysis of the results of Ki-67 expression (Right, n=5). **(E, F)** Analysis of expression of Perforin and Granzyme B of CD8^+^ T in the lymph nodes from the mice by flow cytometry (Left). Quantitative analysis of expression of Perforin and Granzyme B of CD8^+^ T cells in the lymph nodes (Right, n=5). **(G, H)** Analysis of expression of Perforin and Granzyme B of CD8^+^ T cells in the spleens of the mice by flow cytometry (Left). Quantitative analysis of expression of Perforin and Granzyme B of CD8^+^ T cells in the spleens (Right, n=5). All data in this figure are presented as means ± SD. The survival data of the mice were analyzed by the log-rank test and other results were analyzed by ANOVA.

To further investigate whether CD4^+^ T_LEX-CD8086_ cells can work therapeutically on established tumors as a vaccine approach, the therapeutic effect of CD4^+^ T_LEX-CD8086_ cells was evaluated in tumor-bearing mice. For that purpose, L1210 cells were s.c. pre-inoculated in mice on Day 0, when tumors were palpable, the mice were injected with PBS, LEX-null, LEX-CD8086, CD4^+^ T_LEX-null_ cells, and CD4^+^ T_LEX-CD8086_ cells thrice (**Figure 6A**). The median survival days (MSD) of mice treated with CD4^+^ T _LEX-CD8086_ cells was 30 days, notably longer than that of the mice treated with CD4^+^ T_LEX-null_ cell (24 days), LEX-CD8086 (23 days), LEX-null (20 days), and control mice (17 days; **Figure 6B**). Furthermore, we also monitored tumor growth, injection of CD4^+^ T_LEX-CD8086_ cells notably inhibited tumor growth compared to injection with other agents, as shown in **Figure 6C**, at day17, the median tumor volume of mice immunized with CD4^+^ T _LEX-CD8086_ cell was 1209 mm^3^, which was significantly smaller than that of mice immunized with CD4^+^ T_LEX-null_ cell (2048 mm^3^), LEX-CD8086 (2290 mm^3^), LEX-null (2653 mm^3^) and control groups. The expression of Ki-67 of tumor tissues in mice injected with CD4^+^ T _LEX-CD8086_ cell was 53.9%, which was significantly lower than that in the mice injected with CD4^+^ T_LEX-null_ cell (73.5%) and LEX-CD8086 (69.8%) and control (93.4%, **Figure 6D**). These data suggest that CD4^+^ T _LEX-CD8086_ cells exhibited the most potential therapeutic antitumor effects among these vaccinations. Moreover, we also examined the activity of tumor infiltrating CD8^+^ T lymphocytes. As shown in **Figures 6E, F**, the treatment with CD4^+^ T _LEX-CD8086_ cells significantly increased the proportion of Perforin^+^ (17.8%) and Granzyme B^+^ (16.8%) CD8^+^ T cells in the lymph nodes compared to that with CD4^+^ T_LEX-null_ cells (13.6%, 10.3%), LEX-CD8086 (10.2%, 8.87%) and LEX-null (3.78%, 4.45%), whereas the controls did not. Similar results were also observed in the spleen, as shown in **Figures 6G, H**, the proportion of Perforin^+^ (83.0%) and Granzyme B^+^ (22.8%) CD8^+^ T cells in CD4^+^ T _LEX-CD8086_ cell treated mice were notably higher than that in mice with the CD4^+^ T_LEX-null_ cell (73.0%, 10.2%) and LEX-CD8086 (75.4%, 10.1%). However, the above-mentioned vaccinations did not induce any antitumor effects on p388 tumor-bearing mice (data not shown), indicating that CD4^+^ T _LEX-CD8086_ cells can induce potent therapeutic effects *via* promoting CD8^+^ T cells proliferation and differentiation into leukemia antigen-specific CD8^+^ CTL.

**Figure 7:**
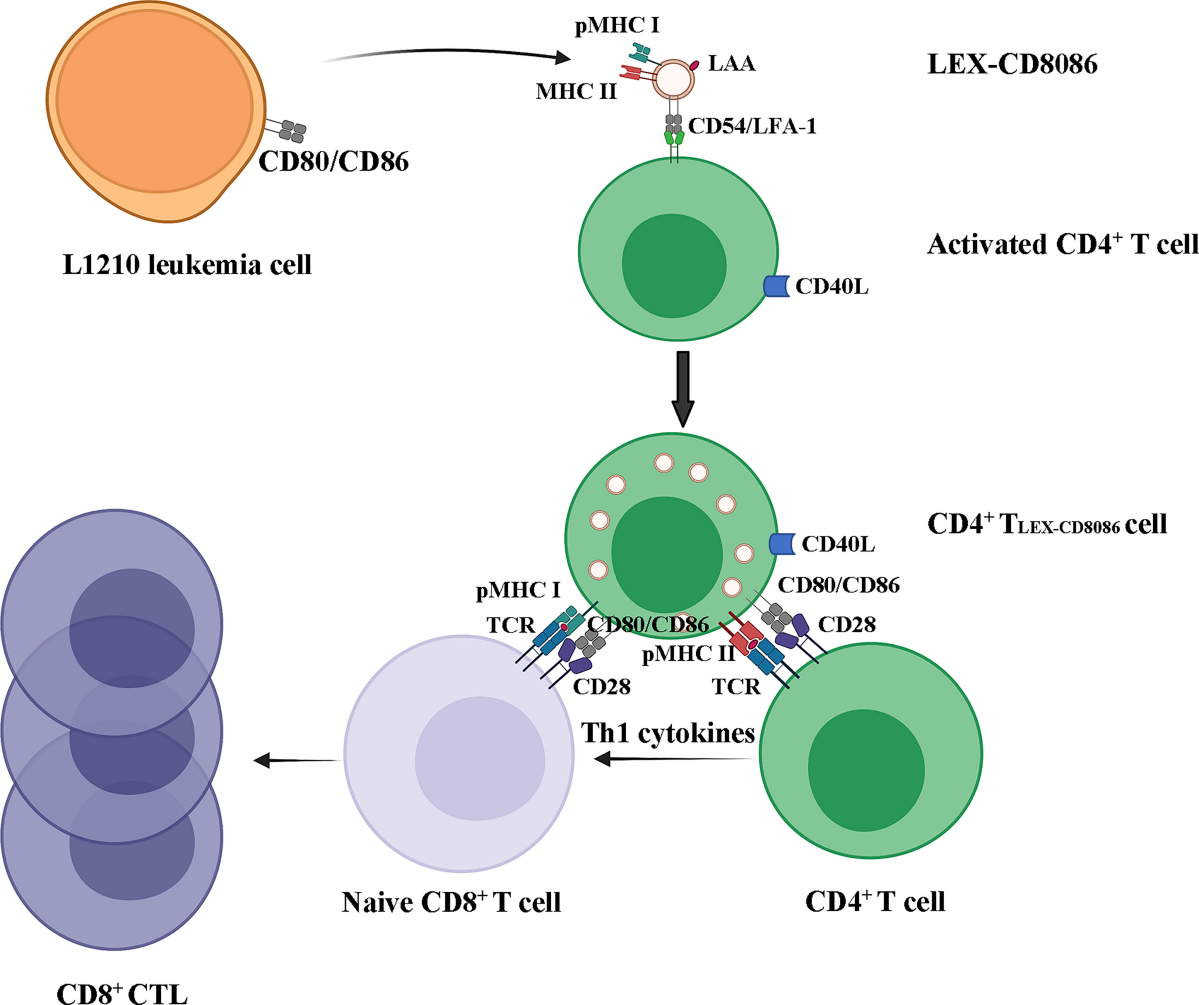
LEX-CD8086 targeting activated CD4^+^ T cells to stimulate a leukemia antigen-specific CD8^+^ T cell response. LEX-CD8086 carrying pMHC I, CD80 and CD86 are fused with activated CD4^+^ T cells and become CD4^+^ T_LEX-CD8086_, and CD4^+^ T_LEX-CD8086_ expressing CD40L and acquired exosomal pMHC I, CD80 and CD86 stimulate antigen-specific, naive CD8^+^ T cell responses *via* IL-2 secretion and CD40L, CD80, and CD86 costimulations.

Taken together, our data suggest that leukemia cells can be genetically modified to upregulate CD80 and CD86 expression in LEXs. LEX-CD8086 can be taken up by activated CD4^+^ T cells and CD4^+^ T cells can acquire exosomal pMHC I and costimulatory molecules from LEX-CD8086 and induce leukemia cell antigen-specific CD8^+^ T cell responses *via* pMHC I/TCR (Signal I), CD40L and CD80,CD86 costimulations (Signal II), and IL-2 secretion (Signal III) (40), **Figure 7**).

## Discussion

TEXs comprise a rich reservoir of the whole panel of tumor-associated antigens (TAAs) and various sets of innate stimulatory molecules that can induce tumor antigen-specific antitumor immunity (26, 41). This property makes TEXs an attractive alternative to tumor vaccines in tumor immunotherapy. Unfortunately, the clinical application of TEXs alone frequently results in unsatisfactory antitumor immune effects, which can be attributed, at least partly, to the limited immunogenicity in TEXs and TEX-induced immunosuppression (32, 38, 42, 43). Therefore, enhancing the immunogenicity of TEXs is the key issue for improving of the efficacy of TEX-based vaccines.

Stimulation of T cells by APCs involves at least two signaling events: one elicited by TCR recognition of peptide-MHC I (pMHC I) and the other by costimulatory molecule signaling (e.g., T cell CD28/APC CD80) (44). Along with other TEXs, LEXs carried leukemia-associated antigens (LAAs) and MHC I/II; they cannot effectively activate both allogeneic and autologous T cells, possibly due to the insufficient expression of costimulatory molecules (45). CD80 and CD86 are the most classical costimulatory molecules interacting with CD28 to activate CD4^+^ T cells (46). Thus, upregulating the expression of CD80 and CD86 in LEXs may be a feasible way to enhance the immunogenicity of LEXs. Notably, our and other studies have shown that the genetic modification of original tumor cells could be a feasible and effective approach to improving the immunogenicity of TEXs and LEXs. Parental tumor cells can be genetically modified to become enriched for tumor antigens, microRNAs, and immune-stimulatory molecules in TEXs and LEXs, to directly cooperate with immune cells or indirectly enhance the antitumor immunity mediated by TEXs and LEXs (26, 35, 47).

In this study, we upregulated the expression of CD80 and CD86 in LEXs through genetically modifying parental L1210 leukemia cells with lentiviruses encoding CD80 and CD86 genes and compared the morphology and typical exosomal proteins on modified LEXs to their unmodified counterpart. As expected, no obvious differences were observed between those two LEXs. Thus, it is easy and feasible to regulate the protein composition of exosomes *via* gene modification without affecting the morphological and biological properties of exosomes themselves. Previous studies have demonstrated that up-regulation of CD80 and CD86 in the tumor vaccine systems can successfully improve the immunogenicity of vaccines due to the effect of costimulatory CD80 and CD86 molecules on tumor immunity (48). Moreover, upregulation of CD80 and CD86 expression in LEXs more effectively promoted DC maturation, CD4^+^ T cell proliferation, and Th1 cytokine secretion, thus inducing a stronger leukemia antigen-specific anti-leukemia CD8^+^ CTL response than LEX alone did (35). In this study, consistent with the previous study, there was more Th1 cytokine secretion detected in the peripheral blood of LEX-CD8086 immunized mice and in the supernatants of LEX-CD8086 targeting CD4^+^ T cell culture, indicating that the LEX-CD8086 induced antigen-specific CTL may be, at least a part, CD4^+^ T cell-dependent. Although all mice immunized with LEX-CD8086 more obviously reduced tumor burden than LEX-null and PBS did, none showed long-term survival, suggesting that the upregulation of CD80 and CD86 expression alone in LEX-based vaccines may still not be sufficient to inhibit leukemia progression. Therefore, further optimization of LEX-based vaccines is still required.

LEXs and LEX-CD8086 as antigen and molecule providers to target APCs may also be an alternative optimization method to improve exosome-based anti-leukemia immunity. APCs can process the antigens and present the antigen peptides in the MHC groove, which strongly facilitates the capture of antigen-peptide-specific CD8^+^ T cells driving their expansion and activation (43, 49). Furthermore, the preferable processing of TEXs in the MHC-II-loading compartment leads to CD4^+^ T helper cells activation (42). Although DCs are the most professional APCs, only a very limited number of DCs arriving at the lymph nodes would interact with many CD4^+^ T cells. CD4^+^ T cells acting as APCs can directly induce a proliferative response among the naïve Ag-specific CD4^+^ T population and also enable the naïve Ag-specific CD4^+^ T to function as APCs, thereby further promoting the antigen-specific CD8^+^ CTL response (40). In addition, the ex vivo expansion of DCs has heterogeneity which is highly dependent on the laboratory conditions, and the preparation of DCs under safety conditions is expensive and time-consuming (50). Compared to DCs, it is much more convenient and stable to produce activated CD4^+^ T cells. The previous study has demonstrated that CD4^+^ helper T (Th) cells can acquire membrane molecules from DC *via* DC activation and act as Th-antigen-presenting cells (Th-APC). These Th-APCs with acquired pMHC I and costimulatory CD54 and CD80 molecules can stimulate tumor-specific CD8^+^ CTL responses and induce antitumor immunity (40). Another study demonstrated that active CD4^+^ T cells with the uptake of ovalbumin (OVA)-pulsed dendritic cell (DC_OVA_)-derived exosome (EXO_OVA_) express exosomal MHC I and costimulatory molecules. These EXO_OVA_-targeted active CD4^+^ T cells can stimulate CD8^+^ T cell proliferation and differentiation into central memory CD8^+^ CTLs, and induce more efficient *in vivo* antitumor immunity than DC_OVA_ (51). However, not as expected, active CD4^+^ T cells with the uptake of LEXs did not induce the expected antileukemia immunity like DEXO-targeted active CD4^+^ T cells did in our preliminary study. We conjecture that the lower immunogenicity of LEXs compared to DEXO due to the expression level of costimulatory molecules may be the main handicap for LEXs-targeted CD4^+^ T cells to induce high-level antileukemia immunity since costimulatory molecule gene-modified LEXs could significantly enhance their antileukemia immunity in our recent study (35). Other studies also demonstrated that uptake of TEXs by DCs might enhance the expression levels of costimulatory molecules and prime immune responses (36–38). Therefore, LEX-CD8086 targeted-activated CD4^+^ T cells may be a better approach to further clinical applications than targeting DCs.

To address whether LEX-CD8086 targeted-activated CD4^+^ T cells (CD4^+^ T_LEX-CD8086_) can induce stronger anti-leukemic immune effects, we examined their anti-leukemia immunity *in vitro* and *vivo*. An *in vitro* study uncovered that the CD8^+^ T cells from the CD4^+^ T_LEX-CD8086_ cell immunized mice differentiated more frequently into CTL effectors and specifically induced more L1210 cell apoptosis than the LEX-CD8086 immunized mice did. Furthermore, by comparing the potential stimulation of CD8^+^ CTL responses and anti-leukemia immunity derived from LEX-CD8086 and CD4^+^ T_LEX-CD8086_ cell vaccine, we proved that CD4^+^ T_LEX-CD8086_ cells induced stronger LAA-specific CD8^+^ CTL responses and antitumor immunity than LEX-CD8086, possibly as a result of lacking APC mediation and less Th1 cytokine secretion in LEX-CD8086. We also compared the ability of LEX-CD8086 and CD4^+^ T_LEX-CD8086_ cells to induce antileukemia immunity *in vivo*, i.e., in prophylactic and therapeutic leukemia mouse models. Our results showed that CD4^+^ T_LEX-CD8086_ cells more effectively attenuated tumor growth and prolonged the survival time of L1210 cell-bearing mice than LEX-CD8086 and CD4^+^ T_LEX-null_ cells did. In addition, our findings indicated that vaccination with CD4^+^ T_LEX-CD8086_ cells induced a potent systemic immune response against leukemia *in vivo*.

It has been demonstrated that CD4^+^ T cells acquiring APC-derived molecules can act as APCs (52, 53), which strongly facilitates the capture of antigen-peptides driving both CD4^+^ Th cells and CD8^+^ CTL expansion and activation, and the acquisition of CD80 from APCs by CD4^+^ T cells plays an important role in retaining CD4^+^ T cell activation in the absence of APCs *via* up-regulation of NF-B and Stat5 (54). In this study, we elucidated the molecular mechanism of the stimulatory effects of the CD4^+^ T_LEX-CD8086_ cells. We demonstrated that the stimulatory effect of the CD4^+^ T_LEX-CD8086_ cells is mediated by Th1 cytokines secretion and its acquired exosomal CD80 and CD86 costimulation. CD4^+^ T_LEX-CD8086_ cells can both act as APCs and Th1 cells, which means that the LAA-specific CD8^+^ T cell response stimulated by CD4^+^ T_LEX-CD8086_ may be both CD4^+^ T cell-independent and CD4^+^ T cell-dependent, whereas the LAA-specific CD8^+^ T cell response stimulated by LEX-CD8086 is mainly CD4^+^ T cell-dependent. Therefore, CD4^+^ T_LEX-CD8086_ cells may provide an alternative EXO-based strategy for leukemia treatment, in which CD4^+^ T cells can easily be harvested from the peripheral blood of a patient and activated *in vitro*. These activated, nonspecific CD4^+^ T cells can acquire the leukemia antigen specificity and induce the stimulatory effect for leukemia antigen-specific CD8^+^ CTL responses after incubation with EXO derived from the leukemia cells in peripheral blood or bone marrow of a patient and thus be used as alternative EXO-based vaccines.

This current model still has some limitations. Mice do not completely recapitulate the immune system of humans. Thus, some differences in response could arise as these results are translated into clinical trials. In addition, we only evaluated CD4^+^ T_LEX-CD8086_ cells in the context of L1210 cells. Whether such an approach would be effective in other tumors remains to be determined. Moreover, although the life span of the mice immunized with CD4^+^ T_LEX-CD8086_ cells was prolonged, they eventually died. Therefore, further studies will investigate at the molecular level how to modulate the system used in this study.

Our data showed leukemia cells could be genetically modified to upregulate CD80 and CD86 expression in LEXs. LEX-CD8086 can be taken up by activated CD4^+^ T cells. LEX-CD8086 transfers its intrinsic LAA and molecules and acquired costimulatory CD80 and CD86 molecules to CD4^+^ T cells, making CD4^+^ T_LEX-CD8086_ cells both Th1 cells and APCs capable of inducing efficient leukemia antigen-specific CD8^+^ CTL response. The *in vivo* CD4^+^ T_LEX-CD8086_ cell stimulation effects on CD8^+^ T cell responses may be CD4^+^ T cell-independent and CD4^+^ T cell-dependent. Therefore, despite some limitations, the present study demonstrates that modified LEXs can directly target CD4^+^ T cells, making CD4^+^ T_LEX-CD8086_ cells a potent anti-leukemia vaccine with multiple antigen targets and providing a new EXO-based vaccine strategy for induction of immune responses against tumors and other infectious diseases.

## Data availability statement

The original contributions presented in the study are included in the article/supplementary materials. Further inquiries can be directed to the corresponding author.

## Ethics statement

The animal study was reviewed and approved by Xinhua Hospital Affiliated to Shanghai Jiao Tong University School of Medicine, Shanghai, China. Written informed consent was obtained from the owners for the participation of their animals in this study.

## Author contributions

The authors declare that they have no competing interests. The concept was conceived by JL, and SH. The overall study design was developed by JL and SH. The experiments were performed by JL, FH, YJ,JZ and JW. The data analysis was performed by JL and FH. The manuscript was written by JL, FH and SH. The research was supervised by SH. All authors contributed to the article and approved the submitted version.

## Funding

This research was supported by the National Natural Science Foundation of China (Grant No. 81470314, 81873435). The datasets used during the current study can be obtained from the corresponding author upon reasonable request.

## Conflict of interest

The authors declare that the research was conducted in the absence of any commercial or financial relationships that could be construed as a potential conflict of interest.

## Publisher’s note

All claims expressed in this article are solely those of the authors and do not necessarily represent those of their affiliated organizations, or those of the publisher, the editors and the reviewers. Any product that may be evaluated in this article, or claim that may be made by its manufacturer, is not guaranteed or endorsed by the publisher.

## Abbreviations

DEXO, Dendritic cell-derived exosomes; ALL, Acute lymphoblastic leukaemia; AML, Acute myeloid leukemia; OS, Overall Survival; CAR, Chimeric antigen receptor; BiTEs, Bi-specific T cell engagers; ICB, Immune checkpoint blockers; CRS, Cytokine release syndrome; TEX, Tumor-derived exosome; LEX, Leukemia-derived exosome; L1210-null, L1210 leukemia cell transfected with a null vector; L1210-CD8086, L1210 leukemia cell high expressing CD80 and CD86; LEX-null, L1210-null derived exosome; LEX-CD8086, L1210-CD8086 derived exosome; CD4^+^ T_LEX-null_ cell, LEX-null-targeted CD4^+^ T cell; CD4^+^ T_LEX-CD8086_ cell, LEX-CD8086-targeted CD4^+^ T cells; NTA, Nanoparticle Tracking Analysis; MHC-I, Major histocompatibility complex class I; MHC-II, Major histocompatibility complex class II; APCs, Antigen presenting cells; DCs, Dendritic cells; IL-2, Interleukin-2; TNF-α, Tumor Necrosis Factor-α; IFN-γ, Interferon-γ; CTL, Cytotoxic T lymphocyte; TAA, Tumor-associated antigen; LAA, Leukemia-associated antigen.
